# Gastrointestinal Infection in South African Children under the Age of 5 years: A Mini Review

**DOI:** 10.1155/2023/1906782

**Published:** 2023-08-23

**Authors:** Tshepo Mafokwane, Appolinaire Djikeng, Lucky T. Nesengani, John Dewar, Olivia Mapholi

**Affiliations:** ^1^Department of Life and Consumer Sciences, College of Agriculture and Environmental Sciences, University of South Africa, Science Campus, Florida, Johannesburg, South Africa; ^2^Department of Agriculture, College of Agriculture and Environmental Sciences, University of South Africa Science Campus, Florida, Johannesburg, South Africa; ^3^Centre for Tropical Livestock Genetics and Health (CTLGH), Royal (Dick) School of Veterinary Studies, University of Edinburgh, Edinburgh, UK

## Abstract

**Objective:**

To estimate gastroenteritis disease and its etiological agents in children under the age of 5 years living in South Africa.

**Methods:**

A mini literature review of pertinent articles published in ScienceDirect, PubMed, GoogleScholar, and Scopus was conducted using search terms: “Gastroenteritis in children,” “Gastroenteritis in the world,” Gastroenteritis in South Africa,” “Prevalence of gastroenteritis,” “Epidemiological surveillance of gastroenteritis in the world,” and “Causes of gastroenteritis”.

**Results:**

A total of 174 published articles were included in this mini review. In the last 20 years, the mortality rate resulting from diarrhea in children under the age of 5 years has declined and this is influenced by improved hygiene practices, awareness programs, an improved water and sanitation supply, and the availability of vaccines. More modern genomic amplification techniques were used to re-analyze stool specimens collected from children in eight low-resource settings in Asia, South America, and Africa reported improved sensitivity of pathogen detection to about 65%, that viruses were the main etiological agents in patients with diarrhea aged from 0 to 11 months but that *Shigella*, followed by sapovirus and enterotoxigenic *Escherichia coli* had a high incidence in children aged 12–24 months. In addition, co-infections were noted in nearly 10% of diarrhea cases, with rotavirus and *Shigella* being the main co-infecting agents together with adenovirus, enteropathogenic *E. coli*, *Clostridium jejuni*, or *Clostridium coli*.

**Conclusions:**

This mini review outlines the epidemiology and trends relating to parasitic, viral, and bacterial agents responsible for gastroenteritis in children in South Africa. An increase in sequence-independent diagnostic approaches will improve the identification of pathogens to resolve undiagnosed cases of gastroenteritis. Emerging state and national surveillance systems should focus on improving the identification of gastrointestinal pathogens in children and the development of further vaccines against gastrointestinal pathogens.

## 1. Introduction

Gastroenteritis involves inflammation of the gastrointestinal tract, mainly the stomach and small intestines [1], and can result from changes in diet, treatment with antibiotics [2], and infection involving various microbial agents, including viruses, such as rotavirus, adenovirus, and astrovirus [3], enteric bacteria, such as *Escherichia coli*, *Campylobacter*, *Shigella*, or *Salmonella* [4, 5], and parasites, such as *Giardia lamblia* and *Cryptosporidium* [6]. It can either be acute or chronic depending on the duration of the symptoms. Acute gastroenteritis can be treated by oral rehydration therapy, and intravenous rehydration, which should be considered as an alternative [7], whereas chronic gastroenteritis may be a consequence of a severely damaged stomach lining resulting from long-term inflammation [8]. One of the main symptoms of gastroenteritis is diarrhea, which is defined as more than three watery or loose stools excreted per day [9].

The incidence rate for gastroenteritis indicates an annual global prevalence of over 1.7 billion cases of diarrhea, which equates to 2.2 million deaths [10]. Thus, diarrhea is one of the leading causes of death globally, especially in children under the age of 5 years [11] who, in developing countries, are particularly at risk of the effects of diarrhea. According to the World Health Organization (WHO), most deaths related to diarrhea among children occur in Africa and South-East Asia and are often linked to suboptimal nutrition and inadequate hygienic conditions [12]. In South Africa, diarrhea is the third leading cause of childhood mortality [13–15].

An underdeveloped immune system in children [16], malnutrition [17, 18], and immunosuppression [19] are underlying factors for sporadic cases of gastroenteritis. The etiology of moderate to severe acute gastroenteritis varies in different regions of the world and depends on the host, and seasonal, environmental, and demographic factors [17, 20]. There have been improvements in most of these factors in many countries [21] but in South Africa, the risks and impacts of gastroenteritis remain significant. Therefore, there is an increased need for both governmental and private intervention to develop and enforce control measures and policies to manage and prevent infectious gastroenteritis [22]. This mini review will summarize the current epidemiological status relating to parasitic, viral, and bacterial pathogens responsible for gastroenteritis in children from developing countries with special reference to South Africa. Information from this study is important to guide new research efforts and inform policymakers and relevant authorities in establishing and maintaining gastroenteritis control and prevention measures.

## 2. Survey Methodology

The objective of this study was to review published research from studies on gastroenteritis with special reference to children under the age of 5 years to establish key facts and trends of gastroenteritis in South Africa. It includes sections on microbial research around the globe that addresses specific health challenges resulting in gastroenteritis. The literature in this review was identified through searches of ScienceDirect, PubMed, Google Scholar, and Scopus for peer-reviewed articles that were potentially pertinent by assessing their relevance and evaluating whether they were acceptable for review. The literature search was conducted using search terms: “Gastroenteritis in children, “Gastroenteritis in the world”, Gastroenteritis in South Africa,” “Prevalence of gastroenteritis” , “Epidemiological surveillance of gastroenteritis in the world”, and “Causes of gastroenteritis.” The conclusion of this review highlights the immediate and future need for high-quality and effective measures to control gastroenteritis in children under the age of 5 years in developing countries.

## 3. Global Distribution of Gastroenteritis

**Figure 1:**
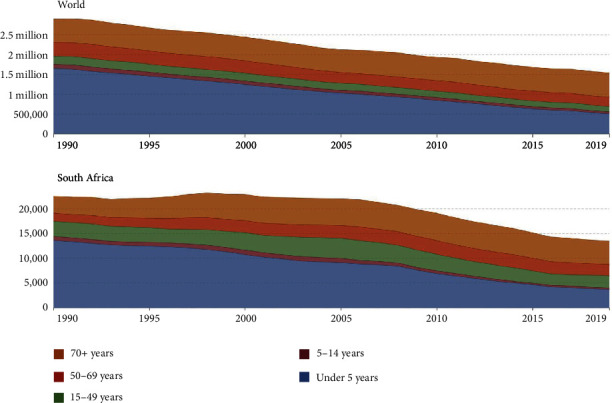
Global and South African mortality rates from diarrheal disease according to patient age [25].

Gastroenteritis is a serious public health concern among children, especially in developing countries in Africa and Asia. Although the mortality rate due to infant diarrhea has decreased over the years resulting from improvements in prevention programs, methods of detection, and treatment methods, it remains a major concern in low-income countries of sub-Saharan Africa [23]. Acute gastroenteritis accounts for 10% of hospitalization and 19% of deaths in children around the globe and around 1.6 million people died due to diarrhea in 2017. As indicated in Figure 1, the highest gastroenteritis mortality rates for 2018 ranged from 20 to 500 per 100,000 individuals and were recorded among the poorest countries of sub-Saharan Africa [24, 25].

Figure 1 reflects a reduction in the rate of gastroenteritis-associated mortality globally and in South Africa over the past three decades. This was mainly due to a reduction in deaths in children under the age of 5 years so that currently one-third of all mortality cases due to diarrheal disease involve children in this age group. However, these mortality figures are mainly generated from under-resourced countries in Africa and Asia and must be treated with caution as being underestimated [35].

## 4. Causes of Gastroenteritis

Rotavirus and *E. coli* are the commonest etiological agents of diarrheal disease and may be spread from person to person, from feces-contaminated water or domestic water, that is, stored or handled unhygienically, from contaminated fish, or unhygienically prepared or stored food [26].

### 4.1. Bacterial Agents

Bacterial pathogens rank second after viruses as frequent causative agents of gastrointestinal disorders in children where they account for 10–20% of acute gastroenteritis cases [27]. Such infection generally affects children of all ages but is most prevalent in toddlers and children under the age of 5 years and is usually diagnosed by clinical features, such as fever and the appearance of blood and mucus in the stool [5]. Infection may be transmitted by direct contact, ingestion of contaminated food, and consumption of water, that is, contaminated with microbes [28]. The resulting dehydration and malnourishment are the two leading causes of mortality from a prolonged infection but with the administration of oral or intravenous rehydration therapy [18], the prognosis for bacterial gastroenteritis tends to be good, especially in developed countries [29]. Various bacteria are responsible for acute gastroenteritis, and most are members of one of the largest bacterial families, the *Enterobacteriaceae*, responsible for a wide range of infections involving fish, plants, animals, and humans [4]. The most common enteropathogenic bacteria are *Campylobacter jejuni*, *Salmonella typhi*, *Salmonella paratyphi*, non-typhoid *Salmonella* spp., *E. coli*, *Shigella* spp., *Yersinia enterocolitica*, and *Vibrio cholerae* [30]. Other notable pathogenic bacteria include enterohaemorrhagic *E. coli* (EHEC) and *Clostridium difficile*. Apart from *Campylobacter* and *Clostridium*, bacterial infections tend to be relatively uncommon in the pediatric population [31]. While several bacterial pathogens have been implicated in diarrhoeal illnesses in other parts of the world, there is a paucity of information concerning the bacterial etiological agents of diarrhea in children in some of the urban and rural areas of South Africa [32]. Some of the bacteria, which cause gastroenteritis are reviewed below.

#### 4.1.1. *Salmonella* species

The *Salmonellae* are a genus of Gram-negative, facultatively anaerobic non-spore-forming bacteria [33]. They are important foodborne pathogens, with the non-typhoidal serovars *Enteritidis and Typhimurium* being the predominant strains [34], as they cause salmonellosis, the most frequent foodborne disease reported in humans who come into contact with contaminated water and food [35]. The pathogenesis of *Salmonella* species involves the invasion of non-phagocytic intestinal epithelial cells following the delivery of a specialized set of effectors involving the type 3 secretion system [36]. The WHO recommends training for food handlers and consumers to keep themselves and their food clean [36] but an estimate of the global and regional burden of invasive non-typhoidal *Salmonella* infections is still needed to inform efforts to prevent/manage these infections [37, 38].

#### 4.1.2. *Campylobacter jejuni*

The *Campylobacters* are Gram-negative, slender, microaerophilic bacteria with a spiral or curved shape [39]. There are 31 different species and 10 sub-species within the genus [39, 40]. *C. jejuni* is a zoonotic pathogen, that is, responsible for 80–90% of the diagnosed cases of *Campylobacter* infections, and represents a major public health concern [40]. Fever, vomiting, abdominal pain, and diarrhea are the most common symptoms of campylobacteriosis [42, 43]. These bacteria colonize the gastrointestinal tract of a wide variety of food-producing animals, such as poultry, cattle, sheep, and pigs [41]. The pathogenesis of *C. jejuni* involves adhesion, gut-wall invasion, colonization, and ultimately the release of toxins [42–44]). The motility of this pathogen is a key factor influencing colonization and survival in the acidic gut environment [45, 46]. The epidemiology of *Campylobacter* spp. is multifaceted due to the wide distribution of the bacteria, their genetic variability, and their interactions with their host [47]. In South Africa, published studies on *Campylobacter* have focused mainly on a relatively small region in the Limpopo Province [48], some were conducted to determine antibiotic resistance[47, 49–51], and only a few studies have reported the prevalence of the pathogen as a public health threat in humans, and specifically in children [52].

#### 4.1.3. *Escherichia coli*

The bacterium *E. coli* is a Gram-negative bacillus of the family *Enterobacteriaceae* and it is the most ubiquitous commensal inhabitant of the gastrointestinal tracts of humans [53]. In addition to gut commensals, this bacterial species includes different pathogenic strains responsible for both intestinal and extra-intestinal disease symptoms. A total of eight *E. coli* pathotypes have been described and, of those, six have been extensively studied and are responsible for intestinal infection. They include enteropathogenic *E. coli* (EPEC), EHEC, enterotoxigenic *E. coli* (ETEC), enteroaggregative *E. coli*, enteroinvasive *E. coli*, and diffusely adherent *E. coli* [54]. Pathogenic *E. coli* is responsible for multiple outbreaks of infant diarrhea, including the *E. coli* strain O104:H4 that produces the Shiga toxin [54] and two pathotypes are known to cause extra-intestinal infections namely: uropathogenic *E. coli* and brain meningitis (suggested as extra-intestinal *E. coli*) [55].

### 4.2. Viral Agents

Several viruses are associated with acute gastroenteritis infection, including rotavirus, norovirus, astrovirus, adenovirus, calicivirus, coronavirus, enterovirus, pestivirus, picobirnavirus, and torovirus [56]. Rotavirus and norovirus are the most common etiological agents of viral diarrhea and account for high morbidity and mortality rates in children. Treatment of viral gastroenteritis has major financial consequences, including hospitalization and physician visits costs [57], and the rate of acute viral gastroenteritis is probably underestimated due to most cases remaining undetected by the healthcare system [36].

#### 4.2.1. Rotavirus

Globally, rotaviruses have been identified as the most common cause of severe acute diarrhea in children under the age of 5 years, accounting for more than 70% of all serious diarrheal cases. The morbidity of rotavirus infection in developing countries is similar to that of developed countries but the mortality of rotavirus disease is higher in developing countries [11]. Rotavirus is a double-stranded RNA virus in the Reoviridae family that consists of various groups, subgroups, and serotypes [58]. At least 15 different serotypes of rotavirus have been identified with five of these (G1, G2, G3, G4, and G9) accounting for the majority of the strains circulating globally [59]. In South Africa, the observed diarrhoeal cases due to rotavirus greatly vary among different age groups with the highest prevalence observed in infants and children up to 2 years in age, with the prevalence decreasing with increasing age of the patient [60]. The possibility of reinfection with rotavirus exists during childhood but its severity decreases after the first infection and few children experience severe symptoms after the second rotavirus infection [17].

In South Africa, rotavirus was identified as a significant cause of both sporadic and epidemic gastroenteritis in infants [61], and in 2009, an attenuated rotavirus vaccine for infants was included as part of the national immunization program [62]. Infection with rotavirus has been documented to follow a seasonal pattern peaking in temperate climates in winter but occurring throughout the year in the tropics [63]. The incubation period of rotavirus infection is about 1–2 days before symptoms can manifest [64]. The symptomatology of rotavirus infection ranges from fever, nausea, vomiting, and severe non-bloody watery diarrhea to abdominal pain [65]. Diarrhea from rotavirus infection can expedite acute and life-threatening complications, such as dehydration, which may be associated with lethargy, sleepiness, constant irritability, dry mouth, thirst and pale, or blotchy colour on the skin [64]. Asymptomatic cases may also occur, especially through successive infection of individuals where protection from rotavirus infection is conferred following immune seroconversion [66].

#### 4.2.2. Norovirus

Noroviruses are recognized as the principal causative agents of viral diarrhoeal outbreaks in both adults and children within closed communities, such as nursing homes, schools, military populations, athletic teams, and cruise ships [67–70]. Such infections are present throughout the year, despite initially being considered a disease that peaked only in the winter months. It has been estimated that each year, 658 million cases of gastroenteritis requiring hospitalization are due to norovirus infection. Of these cases, 200 million are among children under the age of 5 years, leading to an estimated 50,000 child deaths every year, mainly in developing countries [72]. As in other African countries, relatively little is known about the molecular epidemiology of noroviruses in the rural areas of South Africa [73]. Several factors contribute to norovirus outbreaks, including the high infectivity of norovirus particles, the persistence of noroviruses in the environment, prolonged shedding of the virus from both symptomatic and asymptomatic individuals, and a lack of lasting immunity [74].

Noroviruses are non-enveloped viruses with a single-stranded RNA genome approximately 7.5 kb in length [75]. Noroviruses are genetically classified into five genogroups, GI–GV, with GI and GII strains responsible for most human diseases and outbreaks. Once every two to four years, new strains of noroviruses emerge, which often lead to an increase in disease outbreaks worldwide [71]. Immunity to the virus is not well understood, with most research efforts to understand protective immunity against norovirus having focused on humoral immunity [72, 73].

Despite significant progress towards furthering our understanding of the immune response and its correlates with immune protection following attempts to vaccinate against norovirus infection, many questions remain unanswered, including those relating to the role of host genetic susceptibility, which is associated with the expression of histo-blood group antigens within the intestinal lumen [56, 74]. Currently, there is no vaccine against norovirus but several vaccine strategies, most using virus-like particle (VLP) antigens, are under development and have shown proof of efficacy, the most advanced being the adjuvanted bivalent intramuscular norovirus VLP vaccine [75]. The incubation period for norovirus infection is about 1–2 days and symptoms start manifesting as early as 12–48 hours after exposure and can last up to 3 days [76]. Symptoms are more intense in children compared with adults and include nausea, diarrhea, stomach pain, cramps, and vomiting [71] contributing to dehydration and possible death. Norovirus infection has been reported to be associated with cases, such as necrotizing enterocolitis in neonates, postinfectious irritable bowel syndrome, and chronic diarrhea in immunocompromised people [76].

#### 4.2.3. Adenoviruses

Adenoviruses are classified in the family Adenoviridae, genus *Mastadenovirus*, which contains seven known species, from A to G. They are double-stranded, linear, non-enveloped DNA viruses. The genome length of adenoviruses is about 26–45 kbp and it is characterized by an inverted terminal repeat of 36–371 bp [77]. The genome of adenoviruses is organized into at least 16 clearly defined genus-common genes, including that expressing a polymerase, as well as a set of more variable genus-specific genes usually located near the ends of the genome [78, 79]. It is estimated that there are over 50 distinct serotypes known to cause human infections [80] and the mechanisms that relate to their evolution include the accumulation of punctate mutations, homologous recombination, gene capture, and inter-species transmission [77]. Adenoviruses are ubiquitous in the environment, especially when polluted by human feces or sewage [81]. They are known to infect multiple sites, including the respiratory tract, the eyes, and the urinary tract [82]. Respiratory symptoms include fever, cough, wheezing, and sore throat with diarrhea being a common gastrointestinal symptom [83]. Either symptomatic or asymptomatic cases of adenoviruses occur depending on the immune status of the host or the virus pathogenicity and strain [84]. Adenoviruses have been reported to co-evolve with their host [78].

### 4.3. Protozoan Agents

Intestinal parasitic infections are common in children in developing countries. Protozoan-associated gastroenteritis clinically presents as dysentery associated with fever, bloody diarrhea, a constant need to pass stools, and abdominal pain [85]. To establish infection, parasites have evolved a wide range of mechanisms to evade and manipulate the host's immune response [86]. Enteroparasites responsible for diarrhea includes *Giardia lamblia*, *Cryptosporidium*, and *Entamoeba* spp., which are common within the South African population [87]. Most of these enteroparasites have been identified as neglected tropical diseases [88].

Intestinal protozoans are transmitted via the fecal-oral route and tend to exhibit similar life cycles consisting of a cyst stage, which is characterized by a resistant wall and is excreted along with stools, and a trophozoite stage, which is characterized by an active metabolism, increased uptake of nutrients, motility, and asexual replication [89]. Trophozoites are also excreted along with the stools. Some of the trophozoites develop into cysts instead of undergoing replication. The function of the cyst wall is to protect the organism from desiccation in the external environment, whereas the parasite is dormant before being ingested by the next host [90].

#### 4.3.1. Cryptosporidiosis

Cryptosporidiosis is a diarrheal disease caused by the protozoan parasites *Cryptosporidium parvum* and *Cryptosporidium hominis*. It is the second leading cause of moderate-to-severe diarrhea in children worldwide [91]. *Cryptosporidium* has a very complicated life cycle, with both asexual and sexual stages present in the intestine [92]. Little is known about the pathogenesis of this protozoan parasite, and no safety controls or effective treatments have been successfully developed to combat cryptosporidiosis [93]. Currently, there is no vaccine against cryptosporidiosis, and only one drug (nitazoxanide), with limited efficacy, has been developed. *Cryptosporidium* has been declared a public health concern due to its resistance to standard water chlorine disinfection [94]. The application of molecular tools in the epidemiology of *Cryptosporidium* infections has been used to determine the source of infection in outbreak situations and risk factors of public health significance [95].

#### 4.3.2. Giardiasis

Giardiasis is a diarrheal disease caused by the protozoan parasite *G. lamblia*. It is the most frequently reported intestinal protozoan disease in the world, with about 280 million symptomatic cases and 2.5 million annual deaths each year [96]. Infection may be acquired through direct person-to-person transmission or zoonotic transmission, ingestion of contaminated food, and waterborne transmission [97]. *Giardia* attaches to the gastrointestinal epithelium and induces epithelial cell apoptosis, disruption of tight cell junctions, and increases intestinal permeability [98]. Most individuals who are at risk of this infection are children in day-care settings, child-care workers, institutionalized individuals, travelers in endemic infection areas, people who ingest contaminated recreational water, and immunodeficient individuals [99]. Approximately 50–75% of infected children are asymptomatic [99].

This infection poses a serious public health threat, as it causes intestinal malabsorption and acute diarrhea in children [100]. Understanding the determinants of the outcome of giardiasis is crucial, as the clinical manifestation can range from non-fatal to chronic diarrheal disease [101]. Much research is needed towards understanding the host immune response to the parasite, the pathophysiology as well as the classification of giardiasis due to differences in the genetic assemblages (assemblages A–H), in which only two (A and B) are pathogenic to humans [102].

#### 4.3.3. Amoebiasis

Amoebiasis is an enteric disease caused by the protozoan parasite *Entamoeba histolytica* [103]. It has been reported as one of the most prevalent parasitic diseases worldwide, particularly in developing countries of low socioeconomic status where sanitation and treatment of water may be inadequate [104]. The genus *Entamoeba* contains six species, namely *Entamoeba histolytica*, *Entamoeba dispar*, *Entamoeba moshkovskii*, *Entamoeba bangladeshi*, *Entamoeba poleki*, *Entamoeba coli*, and *Entamoeba hartmanni*. Three of these species (*E. histolytica*, *E. dispar*, and *E. moshkovskii*) are morphologically similar but have different biochemical and genetic features [105]. Of all the species of *Entamoeba*, *E. histolytica* is the only pathogenic species found in the human digestive tract and other organs [106]. Travelers who visit developing countries where amoebiasis is endemic may be infected with this parasite and present with symptoms, including diarrhea, amoebic dysentery, and hepatic liver abscess [107].

Challenges relating to the microscopic diagnosis of amoebiasis arise from the existence of three morphologically identical species leading to the overdiagnosis of *E. histolytica* parasitism [108]. Thus, infection by *E. histolytica* is multifactorial and depends on an interaction between the parasite, host, and microbiota and while much information has been obtained regarding the virulence factors, metabolism, and mechanisms of pathogenicity of this parasite, less is known about its relationship with the host between distinct stages of the disease [109].

## 5. Gastrointestinal Co-Infections

It is increasingly perceivable that pathogen co-infections within the same host are a common occurrence. Thus, chronic infection with pathogens, including malarial parasites, soil-transmitted helminths, *Mycobacterium tuberculosis*, and viruses, such as human immunodeficiency virus (HIV), may affect a quarter of the human population of some developing countries [86].

An infection involving more than one enteropathogen presents difficulties in terms of diagnosis and correct identification, to support appropriate intervention [3]. This is particularly because post-acute illness mortality and growth impairment have highlighted the need for interventions that are more effective than just the management of dehydration and electrolyte disturbance associated with gastroenteritis [110]. Most of the small number of trials of promising novel interventions, such as probiotics and antimicrobials, conducted thus far have taken place in high-income rather than in low-income settings [111].

It is of paramount importance to identify pathogens that may be the potential cause of diarrheal infection in children [112]. Correct intervention in treating gastroenteritis requires specific identification of the pathogen responsible for the infection [112] and this is complicated in cases of co-infection involving multiple bacterial and viral pathogens that may be symptomatic or asymptomatic [113]. Gastric co-infections result in numerous deaths in children, especially in developing countries [114]. The use of quantitative polymerase chain reaction (PCR) to re-analyze stool specimens collected from children in eight low-resource (developing) settings in Asia, South America, and Africa provided insights into gastric coinfection [17]. These authors reported improved sensitivity of pathogen detection to about 65%, and that viruses were the main etiological agents in patients with diarrhea aged from 0 to 11 months but that *Shigella*, followed by Sapovirus and ETEC, had a high incidence in children aged 12–24 months. In addition, these authors reported co-infections in nearly 10% of diarrhea cases, with rotavirus and *Shigella* being the main etiologies together with Adenovirus, EPEC, *Clostridium jejuni*, or *Clostridium coli*.

As indicated, it is difficult to link gastroenteritis to a specific etiological agent, particularly as individuals who are co-infected by multiple groups of pathogens are often considered to be in poorer health than people who have single infections [115]. Thus, co-infections increase the potential for clinical complications and increased treatment costs, and studies intended to investigate the role of co-infections with enteric pathogens in people with acute diarrhea are now urgently required [116]. An understanding of the role of co-infection with enteric pathogens should be prioritized in global health programs to improve the treatment of acute diarrhea in children [117]. Thus, it is important to conduct studies that examine co-infections and correlate co-infection with patient age, gender, and disease severity to optimize the treatment of infectious diarrhea. Such studies are made more complicated as viral and bacterial intestinal pathogens could affect either the same or different regions of the gut [118].

## 6. Infectious Gastroenteritis in Children

As demonstrated in the Global Burden of Disease [119] and from the Global Pediatric Diarrhea Surveillance Network [120], acute infectious gastroenteritis is a common disease. Mortality due to acute gastroenteritis has decreased but infectious gastroenteritis still ranks among the highest causes of child and adult morbidity in developing countries in Asia and Africa [121]. Studies suggest that viruses have replaced bacteria and parasites as the most common pathogens responsible for acute diarrhea [122]; however, in the South African population, bacteria and parasites are still common pathogens responsible for diarrhea [87].

## 7. Management of Gastrointestinal Infections in Children

The most threatening complication in acute gastroenteritis is dehydration. Research has shown that lactose-free formulas can be considered in the management of acute gastroenteritis in hospitalized children below the age of 5 years [123]. The basis of first-line treatment for all children with acute dehydrating gastroenteritis involves the administration of oral or intravenous rehydration solution [124]. Anti-diarrheal medications are generally prohibited in children with acute gastroenteritis because they delay the elimination of infectious agents from the gut [57]. Probiotics are known to be safe and do not interact with medications [125]. Probiotics work by degrading and modifying dietary antigens and balancing the anti-inflammatory response of cytokines and their use may be more beneficial in balancing the immune response against foreign antigens in children with gastroenteritis, as they do not colonize the gastrointestinal tract, which are eliminated within 1–2 hours after ingestion.

## 8. Prevention of Gastroenteritis

Acute gastroenteritis infection may be reduced through implementing personal and food hygiene programs, use of sanitized water, vaccination against potential viruses and bacteria, and breastfeeding and appropriate nutrition. A meta-analysis of 30 studies revealed that improved hand hygiene reduced the incidence of gastrointestinal illness by 31% (95% confidence interval). The use of washing with regular soap was most beneficial, and antibacterial soap provided little additional benefit. Exclusive breastfeeding for four months and partial breastfeeding thereafter is associated with lower rates of acute gastroenteritis in the first year of life and decreased rates of hospitalization from diarrheal disease [126]. Antibodies in human milk contribute a small part of the infant's immune protection, with the intestinal microbiome, prebiotics, probiotics, mucosal immunity, nucleotides, and oligosaccharides assuming greater roles [127].

Another method used to prevent gastroenteritis involves vaccination. One of the first rotavirus vaccines (Rotashield) was removed from the market because it was associated with an increased risk of intussusception, with an incidence of one in 10,000 infants [128]. There are currently four live attenuated rotavirus vaccines, namely, Rotarix™ (monovalent human vaccine), Rotateq™ (pentavalent bovine-human reassortant vaccine), Rotavac®, and Rotasiil™ that have been pre-qualified by the WHO [129]. These vaccines are not associated with an increased risk of intussusception and have strong safety records based on extensive studies, including randomized clinical trials showing that their use reduces the severity of the disease [130]. A norovirus vaccine is currently undergoing clinical trials and studies have shown a robust immune response and good tolerability in adults and children [75]. A cholera vaccine has also been prequalified by the WHO and studies on the efficacy and effectiveness of the vaccine have proven to be preventive for cholera [131].

## 9. Diagnostic Methods

According to the WHO, 86% of the population in developing countries spends roughly $6 per capita per year on medical devices (diagnostic equipment or treating instruments) compared with those in developed countries who spend $290 per capita per year on medical devices. Diagnostic testing of patients with apparent gastroenteritis is guided by clinical assessment [50] but differential diagnosis of the causative pathogens based on clinical symptoms may be difficult due to similar presentations [132]. Current methods used for gastrointestinal diagnostics include clinical examination, where causative agents are detected from stool using microscopy, antigen detection, plate culture, and molecular diagnostics. Furthermore, diagnostic evaluation may be required in the case of patients presenting with severe illness or severe dehydration [133].

Molecular approaches have increasingly brought to light significant viral, parasitic, and bacterial enteric pathogens and their virulent traits [112]. Recently, sequence-independent amplification techniques linked to next-generation sequencing platforms (which are culture-independent) have led to the discovery of several novel infectious pathogens [134]. However, due to a lack of infrastructure, well-trained technicians, and funding to support proper medical treatment for patients, developing countries might find it difficult to implement such diagnostic technologies that have been successfully applied in developed countries [133]. It is, therefore, critical to develop effective platforms for infectious disease diagnosis for use in resource-limited settings.

### 9.1. Microscopy

The use of microscopy for the detection of gastroenteritis is based on the direct visualization of entire bacterial/parasitic particles from the stool of infected patients. It employs phenotypic tests, such as Gram staining, bacteriological culture, and recording of colony characteristics and these plus biochemical tests form the mainstay of laboratory diagnosis, in many developing countries [131]. Other possible screening tests, such as the fecal leukocyte test and the use of fecal lactoferrin markers in identifying intestinal inflammation, can also be used to diagnose diarrheal diseases [136]. This method is advantageous in that it is readily available with minimal instrumentation needed; however, it is costly, and its sensitivity is reported to be less than 60% [137]. Detection limits for *Cryptosporidium* using microscopy were reported in the range of 1 × 10^5^ microorganisms per milliliter of watery stool [138], and this makes the method highly specific but not very sensitive.

### 9.2. Serology

Laboratory diagnosis of gastroenteritis is made by the detection of pathogen antigen or nucleic acid in fresh stool samples obtained during acute illness [139]. Antigen detection tests generally have a high sensitivity and specificity ranging between 90% and 95%. Enzyme-linked immunosorbent assays (ELISA) allow for the rapid detection of pathogens in fecal specimens, although they are less sensitive than molecular-based PCR tests [140]. However, their ease of use, quick turnaround time, and affordability make them useful as an initial tool for confirming gastrointestinal pathogens, such as the etiology in outbreak settings [141]. An indirect ELISA to titrate antibodies is of limited usefulness to distinguish between an acute or previous infection as antibodies are not detectable in an active infection until at least a week into the infection and will remain detectable in individuals for years [142]. Other limitations of ELISA includes cross-reactivity between heterologous antigens, which can lead to false positive results [143].

### 9.3. Molecular Diagnostics

Recent developments in the field of molecular diagnostics have changed the way laboratory diagnosis of enteric infections has been carried out [144]. These include commercially available nucleic acid amplification tests (NAAT) that have focused on the detection of either a single pathogen or multiple pathogens in a multiplex assay format [145]. The availability of highly sensitive PCR-based diagnostic panels for the detection of multi-enteric pathogens has revolutionized testing in clinical laboratories [146]. Some NAAT-based technologies provide improved alternatives in the diagnosis of infectious gastroenteritis caused by a wide range of pathogens and in overcoming some of the difficulties faced in the traditional microbiological and culture-based methods [144]. Thus, PCR has become a gold standard in cases of gastrointestinal pathogenic infections [147].

One of the crucial elements for the implementation of a systematic response to infectious disease is a rapid and sensitive assay for pathogen diagnostics [148]. In this regard, the high throughput, sensitivity, and specificity of molecular-based testing allow rapid diagnosis and better management of gastrointestinal infections [144]. New multi-pathogen tests have been developed to increase testing capacity by allowing for the identification of multiple bacteria, viruses, and parasites in a single test that produces results within hours [146]. Recently, standard sequencing techniques or next-generation sequencing (NGS) have been used in pathogen diagnosis and have proven to have the added advantage of comprehensive genomic coverage[134, 135]. Third-generation sequencing technologies have also been introduced with increased sequencing speed and include single molecule real-time sequencing, nanopore sequencing, and complete genomics sequencing [149].

## 10. Risk Factors Associated with Gastroenteritis

It is important to understand risk factors associated with gastroenteritis as it can aid in improved strategies of prevention and control. Studies have previously identified risk factors for diarrhea, such as including younger age, poor sanitation, weakened immune system, seasonal patterns, and improper water storage [150–153].

**Figure 2:**
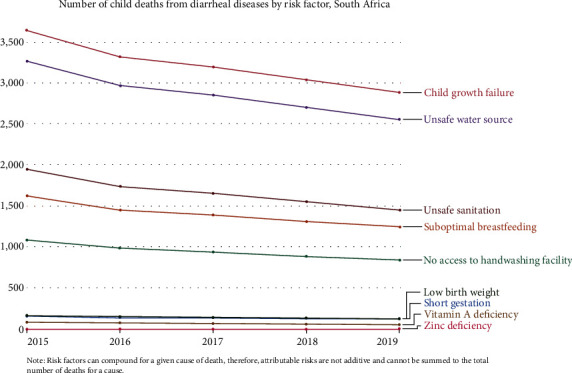
Risk factors associated with diarrheal deaths in African children [25].

Water sources, such as rivers, have no barrier or structure to protect the water from contamination. Thus, river water is easily contaminated with microbes and may not be properly treated. Diarrhea may follow ingestion of such water. In African countries, including South Africa, people often obtain water from communal taps, which are presumed to be clean. Unfortunately, the piped water often comes from poorly maintained and aging water treatment plants using infrastructure that leaks and may be contaminated with biofilms [111]. It is, therefore, important to improve handwashing practices, have a properly maintained water supply, and have proper waste disposal. Some of the factors associated with the ingestion of poor-quality water leading to gastrointestinal infection leading to the death of African children are indicated in Figure 2.

In South Africa, the healthcare system is faced with a persistent problem of gastric infection acquired from within communities or from hospitals (nosocomial infection) [154]. As indicated in Table 1, various gastroenteritis studies have been conducted in South Africa using various study designs and different study populations and sizes. Most of these studies used only established methods of detection, such as culture and PCR, to only detect the most common pathogens, such as *Campylobacter* spp., *E. coli*, *Shigella*, rotavirus, norovirus, adenovirus, and *Cryptosporidium*. In addition, a few of these studies looked at regional comparisons that are important to meet current global initiatives agendas, such as sustainable development goals. In 2021, stool samples were collected from children under the age of 5 years who presented with gastroenteritis at hospitals in Gauteng, South Africa. The specimens were submitted to a commercial pathology laboratory for diagnosis and the specimens tested positive for enteric protozoan parasites and pathogenic bacteria as indicated in Table 2 (Data was obtained from SA Lancet laboratories, 2021, 2021).

## 11. The Economic Importance and Distribution of Gastroenteritis

Gastroenteritis accounts for millions of deaths each year in young children, mostly in developing countries, including South Africa [14]. The Global Burden of Disease study estimated that diarrhea was a leading cause of death, resulting in 1.7 million deaths in 2016 [11]. In addition to the burden of disease and death in children, treatment costs can place a significant financial strain on households and healthcare systems alike [166]. A study in Malawi found that there is a risk of impoverishment due to severe episodes of diarrheal disease [167].

Understanding the global economic burden of gastrointestinal disease is critical. The number of deaths, hospitalized patients, general practitioner consultations, and cases occurring in communities can be described in terms of a disease pyramid (pathogens, the environment, and society), which provides a more complete picture of the burden of illness [168]. Decision-makers, such as funders, policymakers, public health officials, and product developers, need more economic information to determine where gastroenteritis should be situated on their list of priorities and how much time, effort, and resources to invest. Moreover, without updated information on the worldwide distribution of the economic burden, there may not be enough evidence as to where and to whom should resource and efforts be directed [22].

## 12. Conclusions and Perspectives

Diarrheal diseases remain a threat to global public health, especially in the case of children in developing countries where access to quality healthcare is limited, and the costs of such care are prohibitive. The mechanisms underlying gastrointestinal diseases are complex and present substantial research challenges. Recently, enteric viruses were reported to replicate in the salivary glands, and the infection is spread via saliva and reaches titres comparable with those in the gastrointestinal tract [169]. The traditional methods for the detection of pathogens, such as filtration, tissue culture, microscopy, and serology, are powerful [170], and these traditional techniques are now accompanied by molecular methods such, as PCR and DNA sequencing [171]. Furthermore, the pace of technological advances and related innovations in microbial genomics has increased [172] so that genome-wide association studies, in which genotyping information is utilized to systematically investigate the genetic basis of phenotypic diversity, are becoming one of the most successful and widely used approaches to assist in overcoming the problems associated with diagnosis [173]. Metagenomics analysis, based on NGS, is another technique that holds promise as a diagnostics tool and makes possible simultaneous detection and genomic characterization of all microorganisms present in a sample [174]. However, these expensive and sophisticated techniques may not readily be available in developing countries, which can be a limitation in the application of the techniques.

### 12.1. Rationale for the Review

High morbidity and mortality rates in developing countries are attributable to diarrheal diseases [175]. To control the transmission and virulence of infectious agents that cause diarrhea, the United States and other developed countries have kept as a priority proper sanitation in terms of maintenance of a clean water supply, good nutrition, cleanliness, and appropriate disposal of human excreta [176]. This is unfortunately not the case in developing countries, such as South Africa, where there is a critical need for improved water, sanitation, and hygiene [175]. Most of the common etiologies of gastrointestinal infections are viruses, bacteria, and protozoans. Currently, there are only four rotavirus vaccines in the viral domain that are licensed for the prevention of diarrhea, and a few bacterial vaccines are prequalified by the WHO. This emphasizes the need to understand the relationship between causative agents of gastroenteritis and factors affecting its severity to enhance the management of the disease and drive vaccine design.

### 12.2. The Audience Intended for This Review

Diarrhea is a leading cause of morbidity, hospitalization, and mortality in children worldwide, and an estimated 2.5 million deaths are recorded annually in children under the age of 5 years, mainly in developing countries in Africa and Asia. This review is intended to increase knowledge regarding gastroenteritis, particularly for South Africans, many of whom live with HIV and malnutrition. In addition to informing clinical staff as well as healthcare and social workers, such knowledge will benefit government health authorities and the public, caregivers of infants and young children, and remind them of the seriousness of gastroenteritis, particularly regarding potentially fatal paediatric rotavirus infection, as well as the impact of gastric infection on South Africans who may be immunocompromised or malnourished.

## Figures and Tables

**Table 1:** Prevalence of gastrointestinal pathogens in South Africa (mini review).

Regions	Study design	Sample size	Pathogens	References
Vhembe District and Limpopo Province		100	*E. coli*, *C. jejuni/Campylobacter coli*, *Salmonella*, *Shigella Plesiomonas shigelloides*, and *V. cholerae* rotaviruses	[155]
Eastern Cape Province		72	Hepatitis A virus, enteroviruses, and rotaviruses	[156]
Gauteng Province		150	Noroviruses GI and GII	[157]
Eastern Cape Province	Case–control	34	Actinobacteria, Proteobacteria, *Firmicutes*, and *Bacteroidetes*	[158]
KwaZulu-Natal Province			Enteroviruses and enteroviruses	[159]
Eastern Cape Province	Cross-sectional	1009	*Ascaris lumbricoides*, *Trichuris trichuira*, *Schistosoma mansoni*, *Schistosoma haematobium*, and *Taenia* spp.	[160]
Vhembe District and Limpopo Province	Cross-sectional	237	*E. coli*, Shigella, adenovirus, norovirus, and rotavirus	[161]
KwaZulu-Natal Province	Case–control	188	Norovirus	[162]
Limpopo and Gauteng provinces		516	*G. lamblia*	[163]
Northwest Province		505	Rotavirus and norovirus, *Campylobacter* spp. *Arcobacter*, and DEC *C. jejuni*, *C. coli*, *Campylobacter upsalensis*, and *Arcobacter butzleri*	[164]
Gauteng Province		205	Rotavirus, norovirus, adenovirus, sapovirus, and astrovirus	[165]

**Table 2:** Identification of bacterial and parasitic pathogens responsible for gastroenteritis in South African children in 2021.

Pathogen	Percentage (%)
Diarrheagenic *E. coli*	25.1
*Clostridiotes difficile*	11.0
*C. jejuni/coli*	7.3
*Salmonella* spp.	5.0
*Cryptosporidium* spp.	1.9
*G. lamblia/intestinalis*	1.8
*Yersinia enterocolitis*	1.0
*Shigella*	0.4
*E. histolytica*	0.2
*P. shigelloides*	0.2
Non-cholera *Vibrio* spp.	0.2
*Aeromonas* spp.	0.2
